# Probiotic Administration Contributes to the Improvement in Intestinal Dysregulation Induced by Allergic Contact Dermatitis

**DOI:** 10.3390/microorganisms13051082

**Published:** 2025-05-07

**Authors:** Eduardo Mendes, Evelyn Roxana Perez Umana, Daniel Di Pace Soares Penna, Fernando Augusto de Oliveira, Leandro Nascimento Lemos, Willian Rodrigues Ribeiro, Mateus Barbosa Casaro, Mariana Lazarini, Valéria Maia Oliveira, Caroline Marcantonio Ferreira

**Affiliations:** 1 Institute of Environmental, Chemistry and Pharmaceutical Sciences, Department of Pharmaceutics Sciences, University Federal de São Paulo, Diadema 04021-001, Brazil; eduardo.mendes@unifesp.br (E.M.); umana.evelyn@unifesp.br (E.R.P.U.); will.ribeiro181@hotmail.com (W.R.R.); mateuscasaro@gmail.com (M.B.C.); azarini@unifesp.br (M.L.); 2 Division of Microbial Resources, Multidisciplinary Center for Chemical, Biological and Agricultural Research (CPQBA), State University of Campinas (UNICAMP), Paulínia 13148-218, Brazil; d271111@dac.unicamp.br (D.D.P.S.P.); vmaia@cpqba.unicamp.br (V.M.O.); 3 Cellular and Molecular Neurobiology Laboratory (LaNeC), Center of Mathematics, Computing and Cognition (CMCC), Federal University of ABC, São Bernardo do Campo 09280-560, Brazil; oliveira.fernando@ufabc.edu.br; 4 Ilum School of Science, Brazilian Center for Research in Energy and Materials (CNPEM), Campinas 13083-100, Brazil; lemosbioinfo@gmail.com

**Keywords:** dermatitis, microbiome, allergy, intestine

## Abstract

Recent studies have emphasized the impact of gut microbiota on skin health, but the reverse, how skin diseases affect gut homeostasis, has received less attention. Allergic contact dermatitis (ACD), a common skin disorder affecting one in four people worldwide, can be accompanied by intestinal disturbances. To explore this, we used an experimental model of ACD to investigate the intestinal changes induced by the disease. Parameters assessed included intestinal microbiota, short-chain fatty acids (SCFAs), gene expression related to intestinal permeability, inflammatory cytokines, and mucus production. To evaluate potential therapeutic interventions, the probiotic *Bifidobacterium longum* strain BB536 was administered via gavage, starting 10 days before dermatitis induction and continuing until the last day of disease induction. ACD caused alterations in the composition of intestinal microbiota compared to naïve mice but did not affect SCFA production. The probiotic altered microbiota composition and increased acetate production in dermatitis-induced mice. ACD decreased the gene expression of *TjP1*, *ATHO1*, and *MUC2*, while probiotic treatment restored *TjP1* and *ATHO1* to normal levels. The cytokine IL-6 increased in the ACD group compared to naïve mice, whereas IL-10 decreased; probiotic treatment also restored these levels. Intestinal mucus production, affected by ACD, was partially restored by probiotic treatment. The findings suggest that probiotics could be a therapeutic strategy to prevent intestinal issues caused by skin diseases.

## 1. Introduction

Skin diseases, such as dermatitis and psoriasis, are quite common worldwide [1,2,3,4]. Recently, numerous scientific studies have investigated the importance of gut microbiota in skin health, referring to this relationship as the skin–gut axis. Many of these studies emphasize the critical role of probiotics in treating skin diseases [5,6,7]. For instance, many clinical studies have used *Bifidobacteria* and *Lactobacillus* to prevent or treat skin diseases such as dermatitis and psoriasis [8,9,10,11]. Moreover, experimental studies have shown that probiotic use can improve skin health, particularly by boosting the immune system and reducing the severity of allergic reactions [12,13,14].

Many studies highlight that intestinal imbalance or dysbiosis can affect the skin by inducing systemic inflammation and triggering skin diseases such as dermatitis. However, little has been investigated regarding how skin diseases, such as dermatitis, may affect the gut. While inflammatory bowel diseases (IBDs) have been associated with cutaneous manifestations such as pyoderma gangrenosum and erythema nodosum [8], the effects of skin conditions on gut health remain underexplored. Similarly, celiac disease is linked to dermatitis herpetiformis and other skin lesions [9]. Additionally, psoriasis is associated with Crohn’s disease [5,6,7,8]. All these skin diseases are also associated with alterations in the gut microbiota [15,16], but it remains unclear whether the skin condition influences the gut.

This study aims to bridge this gap by investigating how the common skin condition, allergic contact dermatitis (ACD), impacts gut health. Specifically, we hypothesize that ACD, through its inflammatory processes, alters the gut microbiota and disrupts intestinal homeostasis. Furthermore, we also examined whether the administration of the probiotic *Bifidobacterium longum strain* BB536 (BL), which is effective in improving gastrointestinal diseases [13,14,15,16,17], could restore gut alterations caused by skin inflammation.

To explore this hypothesis, we used a mouse model of ACD. ACD is an inflammatory skin condition commonly associated with occupational exposures, accounting for about 70 to 90% of all occupational skin diseases [17]. Studies show that continuous exposure to allergens in a specific part of the body can trigger sensitization of mucous membranes in various organs [18,19]. Given that probiotics have been shown to modulate gut inflammation and improve microbiota balance, we sought to determine if BL supplementation could counteract the gut changes caused by ACD.

Therefore, the primary objective of this study was to determine whether ACD influences the gut and if probiotic administration could restore it, preventing future gut inflammatory diseases caused by skin insults. We evaluated intestinal microbiota, short-chain fatty acids (SCFAs), the expression of genes involved in intestinal permeability and mucus, the expression of pro- and anti-inflammatory cytokines, and the production of acidic and neutral gut mucus. Here, we demonstrate that ACD induces gut alterations, and the administration of BL to mice with dermatitis mitigates most of these changes, offering novel insights into this interaction.

## 2. Materials and Methods

### 2.1. Mice

Female Balb/c mice (20 g) were acquired from the Development Center of Experimental Models for Biology and Medicine at the Federal University of São Paulo (CEDEME). The animals were housed in groups of five per cage in a room with controlled lighting and temperature (12 h light/dark cycles, 21 ± 2 °C) with free access to water and food. All experiments involving laboratory animals were evaluated and approved by the Institutional Animal Care and Use Committee of the Federal University of São Paulo under protocol 6889211218. All procedures adhered to Brazilian National Law number 11.794 (Arouca Law), Decree 6.899, and the normative resolutions of the Conselho Nacional de Controle de Experimentação Animal (CONCEA), the federal agency that regulates all research activities involving animal use in Brazil.

### 2.2. Study Design

**Figure 1:**
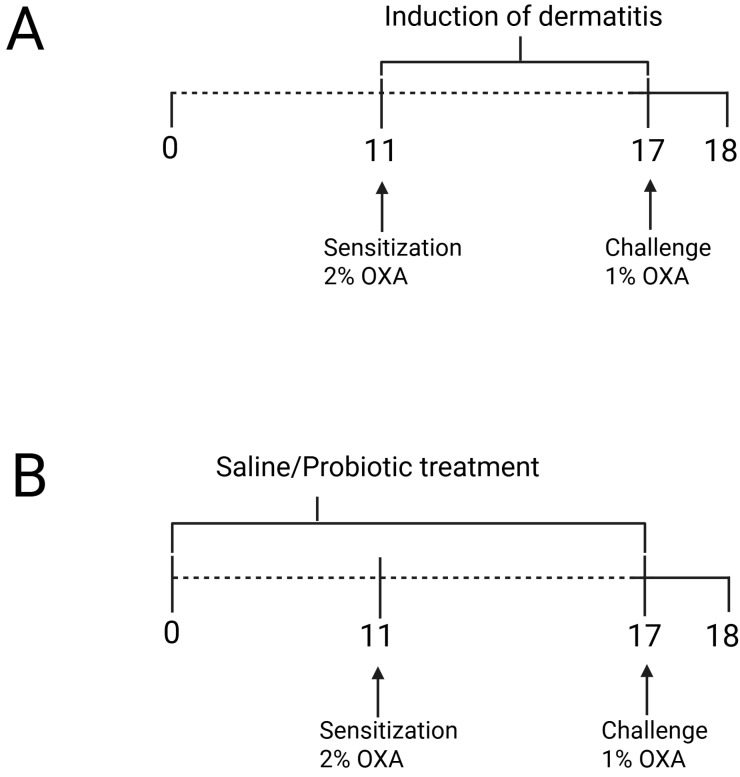
(**A**) Experimental design of oxazolone-induced contact hypersensitivity model and (**B**) probiotic treatment protocol. The animals were sensitized with oxazolone (OXA) on the ear and dorsum, and 7 days later, they were challenged with OXA on the ears. Probiotic treatment was administered via gavage 10 days prior to dermatitis induction and continued until the challenge. Twenty hours after the challenge, the animals were assessed. Figure was generated using Biorender (https://BioRender.com/t08o592, accessed on 15 March 2025).

**Figure 2:**
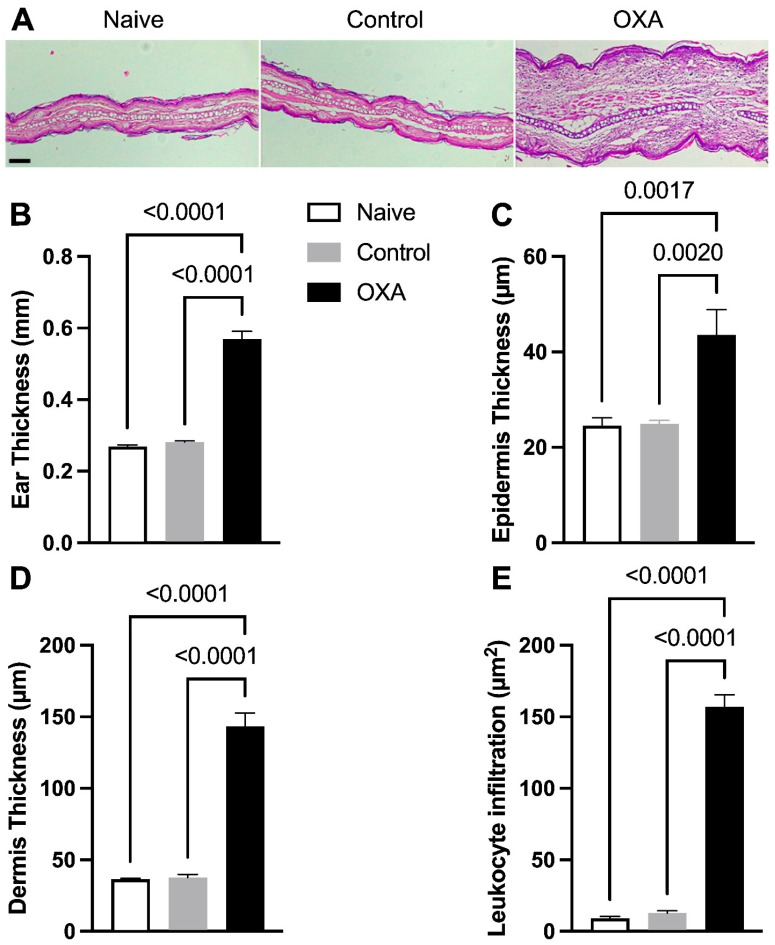
Histological alterations in skin tissue and inflammatory cell accumulation in allergic contact dermatitis. Histological evaluation of skin stained with H&E (Hematoxylin–Eosin). (**A**) Photomicrograph of histological sections of the ear at 40× magnification, scale bar = 0.2 µm. (**B**) Thickness of the right ear (mm). (**C**) Epidermal thickness. (**D**) Dermal thickness. (**E**) Quantification of inflammatory cell infiltration in the skin. Experimental groups: naïve (n = 7), control—animals not induced with experimental dermatitis (n = 7), and OXA—animals subjected to the experimental dermatitis protocol (n = 7). Statistical analysis was performed using one-way ANOVA. Data are presented as mean ± SEM. Statistical significance is considered as *p* < 0.05.

**Figure 3:**
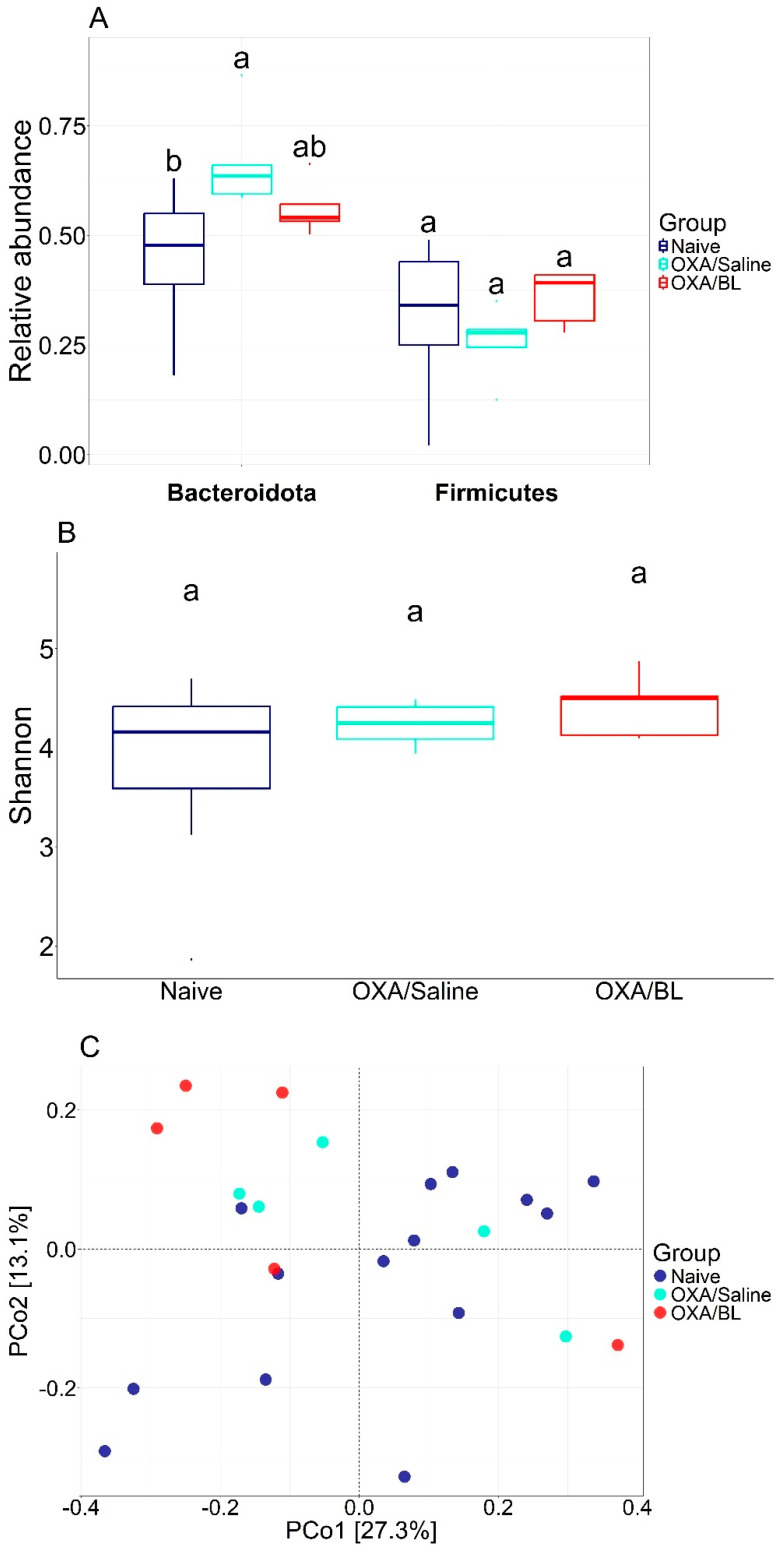
Diversity metrics for different treatments. (**A**) Relative abundance of *Bacteroidota* and *Firimicutes* and statistical difference between each group (ANOVA *p* < 0.05). (**B**) Shannon diversity with statistical test (ANOVA). (**C**) PCoA using Bray–Curtis beta diversity distance. Similar letters indicate statistical significance.

**Figure 4:**
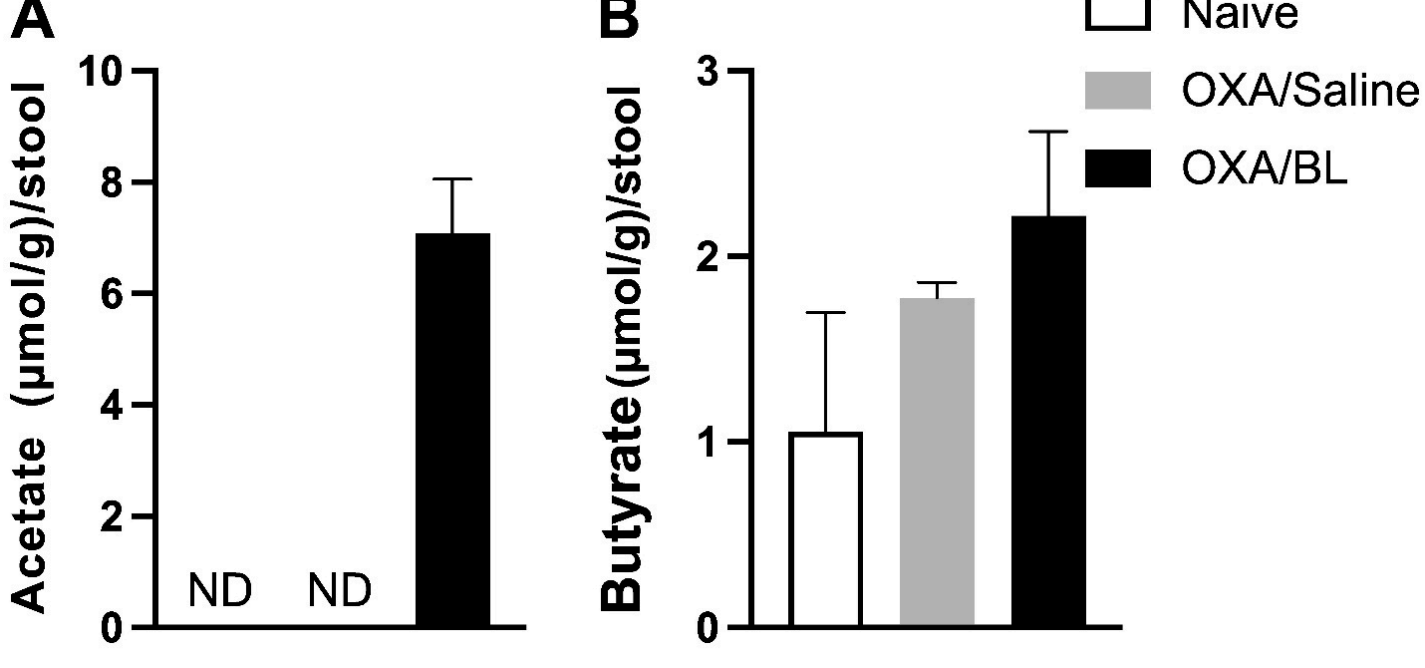
Induction of dermatitis does not alter fecal SCFA levels, while probiotic treatment enhances acetate production in the feces. (**A**) Acetate (µmol/g)/stool. (**B**) Butyrate (µmol/g)/stool. Mice were previously treated with probiotic (OXA/BL; n = 6) or saline (OXA/saline; n = 6), sensitized and challenged with oxazolone. The naïve group was not manipulated (n = 6). Then, 24 h after the challenge, feces were collected and analyzed using gas chromatography for acetate and butyrate. ND: not detectable (below the detection limit). Statistical analysis was performed using one-way ANOVA. Data are presented as mean ± SEM.

**Figure 5:**
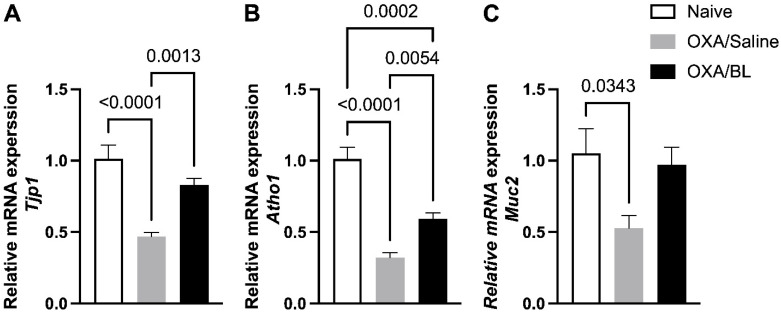
Impact of dermatitis on *Tjp1*, *Atho1*, and *Muc2* gene expression in the intestine: probiotic treatment restores *Tjp1* and *Atho1* expression in mice. Mice were previously treated with probiotic (OXA/BL; n = 7) or saline (OXA/Saline; n = 7), sensitized, and challenged with oxazolone (OXA). The naïve group was not manipulated (n = 6). Then, 24 h after the challenge, the colon was collected and analyzed using RT-qPCR for (**A**) Tjp1, (**B**) Atoh1, and (**C**) Muc2 expression in the intestinal colon. Statistical analysis was performed using one-way ANOVA. Data are presented as mean ± SEM. Statistical significance is considered as *p* < 0.05.

**Figure 6:**
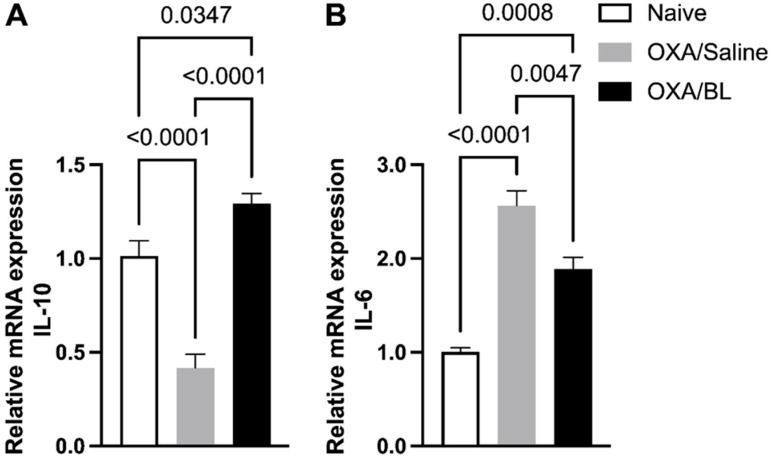
Effect of dermatitis on *IL-10* and *IL-6* gene expression in the intestine: probiotic treatment reverses gene alterations in mice. Mice were previously treated with probiotic (OXA/BL; n = 7) or saline (OXA/saline; n = 7), sensitized, and challenged with oxazolone (OXA). The naïve group was not manipulated (n = 7). Then, 24 h after the challenge, the colon was collected and analyzed using RT-qPCR for (**A**) Il-10 and (**B**) Il-6 expression in the intestinal colon. Statistical analysis was performed using one-way ANOVA. Data are presented as mean ± SEM. Statistical significance is considered as *p* < 0.05.

**Figure 7:**
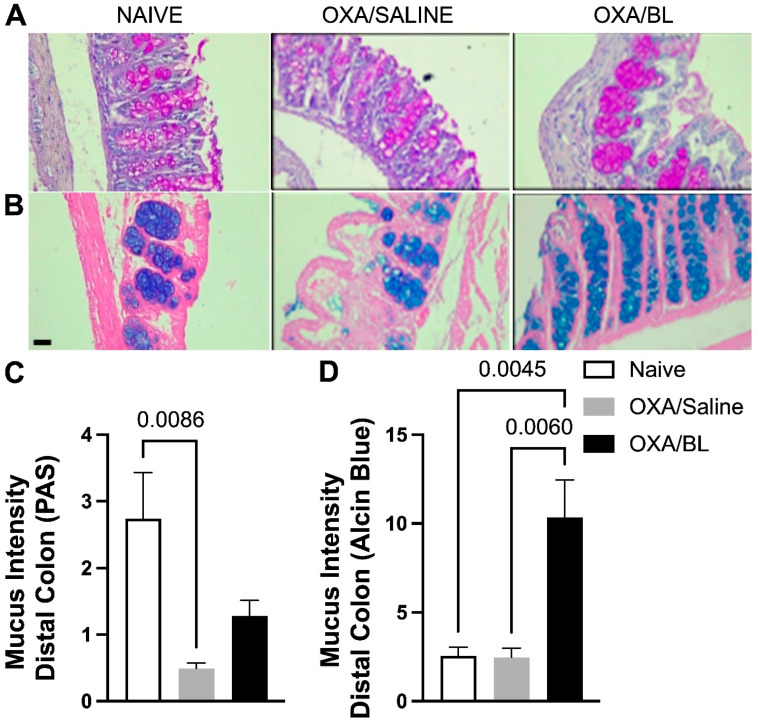
Changes in acidic and basic mucus production in the intestine after skin injury and probiotic treatment. Mice were previously treated with probiotic (OXA/BL; n = 7) or saline (OXA/saline; n = 7), sensitized, and challenged with oxazolone. The naïve group was not manipulated (n = 7). Then, 24 h after the challenge, the colon was collected and prepared for histological staining. (**A**) Photomicrograph of distal colon histology stained with Periodic Acid-Schiff (PAS) and (**B**) Alcian Blue (×60 magnification). (**C**) Basic mucus intensity. (**D**) Acidic mucus intensity. Statistical analysis was performed using one-way ANOVA. Data are presented as mean ± SEM. Statistical significance is considered as *p* < 0.05.

First, we conducted the experimental ACD protocol using OXA, where the control groups were designated as naïve (non-manipulated animals) and control (animals sensitized and challenged with a solution of olive oil and acetone) (Figure 1). Since no significant differences were observed in the skin between the naïve and control groups, we proceeded with the remaining experiments to evaluate the effects of the probiotic. For this, we continued with the naïve, OXA, and OXA BL groups, where OXA BL refers to animals sensitized and challenged with OXA and treated with the probiotic, *Bifidobacterium longum* strain *BB536*, as shown in Figure 2, Figure 3, Figure 4, Figure 5, Figure 6 and Figure 7.

### 2.3. Induction of Allergic Contact Dermatitis

A protocol for inducing dermatitis was carried out by topical application to both ears and back. The hair in these areas of Balb/c mice was removed 24 h before sensitization using an electric clipper (Wahl Clipper, Sterling, IL, USA). The mice were then sensitized with a single application of 50 µL of 2% (*w*/*v*) oxazolone (4-ethoxymethylene-2-phenyl-2-oxazoline-5-one, Sigma-Aldrich, St. Louis, MO, USA) in acetone and olive oil (4:1 *v*/*v*) on the shaved area, whereas the control group was sensitized with a solution of acetone and oil (4:1). (Figure 1A). After seven days, these animals were challenged by the topical application of 10 µL of 1% oxazolone in olive oil (4:1 *v*/*v*) on each ear and 50 µL on the shaved back and evaluated 24 h after the challenge procedure, whereas the control group was sensitized with a solution of acetone and oil (4:1) [13].

### 2.4. Probiotic Cultivation and Administration

*Bifidobacterium longum* strain BB536 was replicated in MRS broth medium and cultured under anaerobic conditions in an anaerobic jar at 37 °C without shaking for 48 h. For the administration of probiotic bacteria, mice received a daily inoculum by gavage of 0.1 mL containing 10^8^ bacterial cells, administered intragastrically daily for 17 days, starting 10 days before sensitization and continuing for 7 days after the start of the experiment (Figure 1B). The OXA/saline group received only phosphate-buffered saline solution for the same period and route of administration.

### 2.5. Evaluation of Inflammatory Infiltration and Morphology of Skin and Intestine

Samples were fixed in 10% formalin, immersed in a 70% alcohol solution for 24 h, then sectioned into 5 µm slices and stained with hematoxylin and eosin (H&E). Epidermal and dermal thickness were measured in 10 images, with 6 measurements per image. Inflammatory infiltration was quantified by counting inflammatory cells in 3 random rectangles per image. Results are expressed as the mean of the areas per slide corresponding to each animal. Gut mucus quantity was assessed by quantifying the color intensity in the colon. Histology was prepared by embedding the samples in paraffin, sectioning them into 5 µm slices, and staining with Periodic Acid-Schiff (PAS) and Alcian Blue. Slides were then photographed using a Leica microscope equipped (Leica Microsystems, Wetzlar, Germany) with Leica Application Suite EZ v. 3.4.0. Image analysis was performed using Image-Pro Plus 4.5 software. The intensity of the staining was quantified, and results were expressed as the ratio between the darkest and lightest colors.

### 2.6. Determination of Cytokine Levels in Colon

Samples were processed using a Polytron machine (Kinematica AG, Luzern, Switzerland) at a constant speed of 30,000 rpm in 1 mL of RIPA lysis buffer solution (50 mM of Tris-HCl pH 7.4, 150 mM of NaCl, 1 mM of EDTA, 1% NP-40, 0.25% sodium deoxycholate) with a protease inhibitor cocktail (Sigma-Aldrich P8340) for every 100 mg of tissue. The supernatant was used to perform the ELISA technique, using RD kits for IL-10, IL-13, IL-33, and IL-6. Readings were taken using a plate reader, and data analysis and acquisition were performed using Gen5 software (version 3.0, BioTek Instruments, Winooski, VT, USA).

### 2.7. Gene Expression Analysis

The expression levels of genes involved in colon tissue homeostasis were evaluated using real-time PCR (polymerase chain reaction) technique. Total RNA was extracted with Trizol reagent (Invitrogen Life Technologies, Carlsbad, CA, USA) and reverse transcribed into cDNA (High-Capacity cDNA Reverse Transcription Kit, Applied Biosystems, Foster City, CA, USA). Gene expression was evaluated via real-time PCR using a Rotor-Gene Q (Qiagen, Hilden, Germany) and SYBR Green as the fluorescent dye (Platinum^®^ SYBR^®^ Green qPCR SuperMix UDG, Invitrogen, Carlsbad, CA, USA). The primer sequences were as follows: *Il-6*: (5′ ACA GCC ACTCACCTCTTCAG 3′, 5′ TCCACCACCTGTTGCTGTATT 3′), *Il-10*: (5′ AAGGAGCCTGGAACACATCCTGT 3′, 5′ AGTTCCCAAGCAGCCCTTCCATTT 3′); *Tjp1*: (5′ CAT TGC TGT GCT CTT AGC GG 3′); *Atoh1*: (5′ GCC TCT GGT CTG GGT TTC AC 3′); *Muc2*: (5′ CAA CTG AAT CCT CGA CGC CT 3′, 5′ GGG AGGG GGA AGG AGT GGA 3′).

### 2.8. Sequencing Processing

16S rRNA amplicon sequence data were processed using the DADA2 software package [20], following the standard protocol outlined in the software manual. In short, amplicon sequencing variants (ASVs) were identified and quantified through five steps: filtering, dereplication, sample inference, chimera identification, and merging paired-end reads [20]. Once the ASV table was generated, we used the naïve Bayesian classifier method proposed by [21] to predict taxonomy identification using the latest version of the SILVA database [22]. Sequences were submitted to the NCBI Sequence Read Archive under the number NCBI PRJNA1225149.

### 2.9. Comparative Analyses

Microbiome [23] and microbiome Marker [24] packages were used to test hypothesis of alpha and beta diversity and to detect potential biomarkers. Firstly, we rarified the asv-table tp 7335 ASVs to avoid bias of depth sequencing levels. The number of ASVs (richness) and Shannon diversity index were estimated using alpha function from microbiome package. Multiple comparisons were performed using one-way ANOVA test. PCoA (Principal Coordinates Analysis) based on Bray–Curtis similarity was generated using phyloseq package [25]. The microbiome packages [26] and microbiome Marker [24] were used to test the hypothesis of alpha and beta diversity and detect potential biomarkers. Firstly, we rarefied the ASV table to 7335 ASVs to avoid bias from depth sequencing levels. The number of ASVs (richness) and the Shannon diversity index were estimated using the alpha function from the microbiome package. Multiple comparisons were performed using the one-way ANOVA test. PCoA (Principal Coordinates Analysis) based on Bray–Curtis similarity was generated using the phyloseq package [27]. The indicator species (ASVs) for each group were determined using the IndVal approach with the multipatt function from the Indic species package for R (v.4.4.3). This approach identifies taxa significantly associated with each group of interest. The IndVal value ranges from 0 (lower association) to 1 (higher association) [28].

### 2.10. Quantification of Short-Chain Fatty Acids (SCFAs)

SCFA measurement was performed as previously described by Ribeiro et al. [29], with adaptations. Briefly, fecal samples (20 mg) were homogenized with 100 μL of distilled water. Then, 40 mg of sodium chloride, 50 μL of 1 M hydrochloric acid, and 300 μL of n-butanol were added. The samples were vortexed for 2 min and centrifuged at 18,000× *g* at 4 °C for 10 min. The supernatant was transferred to chromatographic vials and analyzed using a Shimadzu 2010 Gas Chromatograph system (Shimadzu Scientific Instruments, Kyoto, Japan) with GC Solution software (version 2.41.00, Shimadzu Scientific Instruments, Kyoto, Japan), equipped with a Flame Ionization Detector (FID) and an AOC-20i autosampler, using a 30 m × 0.25 mm internal diameter (I.D.) fused silica capillary Rtx-wax column (Restek Corporation, Bellefonte, PA, USA). A sample volume of 1 μL was injected at 260 °C using a split ratio of 25:1. The oven temperature was initially set to 100 °C and held for 2 min, then increased to 200 °C at a rate of 15 °C per minute and held for 5 min. The FID temperature was set to 260 °C, with hydrogen (H_2_), synthetic air, and nitrogen (N_2_) make-up gas flow rates maintained at 35, 350, and 25 mL/min, respectively. The total runtime for each analysis was 13 min. A calibration curve was constructed to quantify SCFAs, covering a concentration range of 0.015 to 1 mg/mL. Data were expressed as μM.

### 2.11. Statistical Methodology

All data were analyzed using the non-parametric Student’s *t*-test and one-way ANOVA, followed by Tukey’s post hoc test for multiple comparisons, using GraphPad Prism version 8.

## 3. Results

### 3.1. Oxazolone-Induced DCA Increases Skin Thickness and Inflammatory Cell Infiltration

The dermatitis model was developed to induce skin injury in animals to understand its effects on the intestine and whether a probiotic could protect the intestine from these potential effects. Therefore, we first present the skin alterations caused by this model (Figure 2). The results demonstrated that the OXA group showed a significant increase in ear thickness compared to the control group (Figure 2A). Histological analysis supported these findings, revealing increased thickness of the ear (Figure 2B), the epidermis (Figure 2C), and the dermis (Figure 2D), as well as a greater inflammatory cell infiltrate in the ears of the OXA group (Figure 2E). All these data indicate the induction of ACD. Probiotic treatment affected the skin by reducing its thickness but did not affect the infiltration of inflammatory cells (Figure S1).

### 3.2. Effects of Dermatitis and Probiotic Treatment on Gut Microbiota Composition

We analyzed the impact of dermatitis on the intestinal microbiota and observed several alterations, including shifts in bacterial diversity and the abundance of specific bacterial species. Subsequently, we treated the animals with probiotics to determine whether these changes could be reversed. The most abundant phyla were *Bacteroidota*, *Campylobacterota*, and *Firmicutes*, accounting for over 95% of the relative abundance. When comparing the abundance of *Bacteroidota* and *Firmicutes*, there was a significant increase in the relative abundance of *Bacteroidota* in the OXA/saline treatment (*p* < 0.05) compared to the naïve group. Conversely, a tendency for a decrease in *Bacteroidota* was observed when comparing the naïve and OXA/BL groups to OXA/saline, suggesting a potential restoration of *Bacteroidota* composition. A similar pattern was observed for the relative abundance of *Firmicutes*, showing a decreasing trend but not significant (*p* ≥ 0.05) (Figure 3A). Bacterial alpha diversity, based on the Shannon index, was similar between the treatments and did not show a significant difference (Figure 3B) (*p* ≥ 0.05). Beta diversity comparisons indicated that, generally, the gut microbiota composition of the naïve group differed from that of the OXA/saline and OXA/BL groups. However, there was a similar composition between OXA/saline and OXA/BL, indicating a homogenization of bacterial communities (Figure 3C). The identification of potential indicator species followed a similar pattern, with several candidate bacterial taxa associated with changes in bacterial abundances (Table 1 and Figure S3). Members of the phylum *Firmicutes* were present across all treatments; however, the naïve group had *Firmicutes* as the sole indicator, whereas the OXA/saline and OXA/BL groups also included *Bacteroidota*, *Patescibacteria*, and Actinobacteria (only in OXA/BL). The strongest indicators for the naïve group were the *Eubacterium brachy* group (IndVal = 0.91) and the family *Erysipelotrichaceae* (IndVal = 0.88). For the OXA/saline group, the most robust indicators were the family *Muribaculaceae* (IndVal = 0.93) and the genus *Lachnospiraceae NK4A136* group (IndVal = 0.92). In the OXA/BL group, the strongest indicators were an ASV from the order *Clostridia* (IndVal = 0.99) and the family *Lachnospiraceae* (IndVal = 0.96).

### 3.3. The Induction of Dermatitis Does Not Affect the Amount of SCFAs in the Feces, but Probiotic Treatment Increases the Production of Acetate in the Feces

In this study, we evaluated the levels of short-chain fatty acids (SCFAs) in the feces of the naïve, control, OXA, and OXA/BL experimental groups. The primary objective was to determine whether dermatitis affected the production of SCFAs by the intestinal microbiota and whether a probiotic could increase the concentration of these acids. SCFA production was measured using gas chromatography. Our results indicated that acetate was detected only in the OXA group treated with probiotics (Figure 4A). Butyrate was present across all groups; however, there were no statistically significant differences in its production among the groups (Figure 4B). Propionate was not detected in any group. These findings suggest that while probiotics may influence acetate production in dermatitis-induced conditions, they do not significantly affect butyrate levels.

### 3.4. Dermatitis-Induced Intestinal Barrier Gene Disruption Reversed by Probiotic Treatment in Mice

We analyzed whether skin injury could affect the expression of genes associated with the intestinal barrier and cytokines involved in intestinal inflammatory processes. We observed a reduction in the expression of *Tjp1*, *Atho1*, and *Muc2* in the OXA group compared to the naïve group (Figure 5), indicating that skin injuries can affect intestinal gene expression. Probiotic treatment elevated the expression of *Tjp1* and *Atho1* compared to the OXA group (Figure 5A,B). For *Muc2* expression, no significant differences were observed between the OXA group and the OXA group treated with probiotics (Figure 5C), although there was a trend toward increased expression in the probiotic-treated group (*p* = 0.069).

### 3.5. Dermatitis-Induced Intestinal Il-10 and Il-6 Gene Alteration Reversed by Probiotic Treatment in Mice

Additionally, we assessed the expression of cytokines Il-6 and Il-10, revealing an increase in Il-10 (Figure 6A) and a decrease in *Il-6* expression (Figure 6B) in the OXA group compared to the naïve group. Probiotic treatment reversed these effects, reducing *Il-6* expression (Figure 6B), a pro-inflammatory cytokine, and increasing *Il-10* expression (Figure 6A), a regulatory cytokine associated with anti-inflammatory effects. In parallel with the gene expression analysis, we also evaluated cytokines in the intestinal colon homogenate using the ELISA method for Il-6, IL-13, IL-33, and IL-10 (Supplementary Figure S2). IL-10 was not detected in any of the studied groups. We observed that IL-6, IL-13, and IL-33 (Supplementary Figure S2A–C) were not detected in the naïve group but were present only in the intestinal homogenate of animals with dermatitis treated with probiotics, with no differences between these experimental groups.

### 3.6. Impact of Intestinal Mucus Production Following Skin Injury and Probiotic Treatment

To evaluate whether skin injury caused by dermatitis could affect intestinal mucus production, which is crucial for the defense of intestinal tissue, we assessed acidic mucus production in the distal portion of the intestine using PAS staining (Figure 7A) and Alcian Blue staining (Figure 7B). It was observed that the OXA group exhibited a reduction in basic mucus production compared to the naïve group, and probiotic treatment did not restore basal levels (Figure 7A,C). When assessing acidic mucus production in the intestine, no differences were found between the naïve and OXA groups; however, probiotic treatment increased acidic mucus production in animals with dermatitis (Figure 7B,D).

## 4. Discussion

Numerous studies have documented how disturbances in the gut microbiota can influence skin physiology, leading to conditions such as acne and dermatitis [30,31,32]. However, there is a paucity of research exploring whether skin disorders, in turn, impact the gut microbiota and intestinal health. In this study, we investigated the impact of skin injury induced by an ACD model on gut microbiota and intestinal health. Additionally, we explored the effects of BL on the observed gut alterations.

Initially, we implemented an ACD model in mice, which induced morphological changes in the skin, including increased thickness, edema, and an influx of inflammatory cells. Subsequently, we analyzed the gut microbiota of animals with dermatitis and observed alterations compared to naïve animals. The most abundant phyla in the studied groups were *Bacteroidota*, *Campylobacterota*, and *Firmicutes*. Beta diversity analysis indicated that the microbial communities of the gut microbiota of naïve animals differed from those of animals with ACD treated with saline and probiotics. Our findings show that the families *Bacteroidota* (belonging to the phylum *Bacteriodetes*) and Patescibacteria (belonging to the phylum *Patescibacteria*) are common among the groups sensitized and challenged with OXA. While the Bacteroidota group is associated with the production of SCFAs and improved intestinal permeability, *Patescibacteria* is not very well studied, but it can be related to health and disease [33,34,35]. Currently, there is limited evidence directly linking these bacteria to oxazolone (OXA)-induced dermatitis models or ACD in humans. In a previously published study, taxonomic analysis revealed that the non-ACD and ACD mice clustered into two distinct groups, indicating that ACD induced alterations in the gut microbiota structure [36]. Additionally, the same study associates ACD with the relative abundances of *Lachnospiraceae* (belonging to the phylum Firmicutes) and *Desulfovibrionaceae* (belonging to the phylum Proteobacteria), which were higher in the ACD mice compared to the non-ACD mice. In this study, ACD was induced by 2,4-dinitrofluorobenzene (DNFB) [36]. It would be interesting to evaluate whether different chemicals applied to the skin could affect gut microbiota in different ways, as in our study, where ACD was induced with OXA, we did not observe the same results as those seen with DNFB-induced ACD. Studies in children with eczema have shown that microbiome diversity is lower in children with eczema compared to those without it [37,38]. Additionally, *Bifidobacteria* and *Lactobacillus* are more abundant in children without eczema [39,40,41]. These studies differ from ours, likely because they focus on children up to 5 years old and examine a different type of skin allergy, rather than contact dermatitis. However, the OXA group treated with saline and probiotics had common intestinal bacterial phyla. Animals with induced ACD treated with saline showed an increased abundance and predominance of the *Muribaculaceae* family, which is known for producing SCFAs through diet fermentation and mucin glucan degradation. It is also worth noting that probiotic treatment restored bacteria families from the Firmicutes phylum, such as *Lachnospiraceae*, which is also a SCFAs producer [42,43]. This seems relevant since the naïve group showed a predominance of bacteria from the Firmicutes phylum, such as *Ruminococcaceae* and *Erysipelotrichaceae*.

Given the identified changes in bacteria responsible for SCFA production, we proceeded to evaluate these acids in the feces of the animals. Dermatitis does not affect the production of SCFAs such as acetate and butyrate, but probiotic treatment increases the production of acetate. This is interesting, considering that the probiotic used has already been described as an acetate producer [44,45]. It has also been demonstrated that acetate produced by the *Bifidobacterium longum* strain *BB536* during carbohydrate fermentation stimulates the growth of butyrate-producing colon bacteria and promotes the in vitro production of butyrate [46]. However, in this study, we did not observe any differences in butyrate production between the experimental groups, even though the probiotic increased *Lachnospiraceae*, a family known for producing butyrate, as its most widely produced SCFA. This may be due to the timing of fecal sample collection for SCFA analysis or the possibility that butyrate produced was being utilized in greater amounts by enterocytes in the ACD group treated with probiotic, which would prevent the detection of differences in the feces. Additionally, it would be interesting to examine whether there are differences in SCFAs in the serum of the animals, as this was not analyzed in the present study. It is well known that *Bifidobacteria* primarily produce acetate, and in smaller amounts, butyrate and propionate [46]. As a result, this may explain why we did not observe differences in these SCFAs.

It is worth noting that a study using an ovalbumin model to induce allergic dermatitis in mice also found no differences in fecal SCFA levels between mice sensitized on the skin with OVA or saline [47]. However, they demonstrated that antibiotic treatment in mice with ovalbumin-induced allergic dermatitis led to a worsening of clinical signs and a decrease in fecal SCFA production. When the microbiota was restored through fecal administration from healthy mice or probiotic treatment, the effects on SCFA production varied. Notably, propionate and valerate were significantly associated with fecal and probiotic treatments in the AD mouse model [47]. These findings suggest that while skin sensitization alone may not alter fecal SCFA levels, disruptions in the microbiota, such as those caused by antibiotics, can significantly impact SCFA production and disease severity, with potential restoration through microbiota-targeted interventions. These SCFAs, especially acetate, have significant effects on the intestine at the molecular and cellular levels. Acetate is associated with improved intestinal permeability and positively influences the production of mucus by goblet cells, which are responsible for the secretion of mucin, increased production of regulatory T cells, and consequently, the anti-inflammatory cytokine IL-10 [48,49,50,51].

We then evaluated the expression of *Il-6* and *Il-10* genes in the distal colon and observed that ACD promoted a reduction in the expression of the anti-inflammatory cytokine IL-10 and an increase in the expression of the IL-6 gene. However, the probiotic was able to reverse these findings, increasing IL-10 gene expression and reducing IL-6. IL-6 is associated with the development of intestinal diseases such as colorectal cancer and colitis [52,53,54]. Studies have shown that Bifidobacteria can downregulate IL-6 while upregulating IL-10 in the intestine [54,55], especially the bifidobacteria used in this study [56]. IL-10 is a cytokine primarily produced by regulatory T cells (Treg cells), which play a crucial role in controlling various allergic inflammatory responses [57]. When we measured cytokine levels for IL-33, IL-6, and IL-13 in the homogenate of intestinal tissue, the basal group presented cytokine levels below the detection limit. However, in the dermatitis groups, these values were higher. There were no differences between the OXA and probiotic-treated OXA groups, which may be due to differences being present only at the RNA expression level. Here, we do not measure cytokines in the blood, only in the intestine. However, clinical studies generally investigate cytokines and immune cells in the blood of patients. There is evidence in the literature that probiotic intake can modulate IL-10 and Treg cells in the blood of patients with dermatitis. A study showed that 48 adult patients with allergic dermatitis who consumed probiotics (*Lactobacillus salivarius* LS01 and *Bifidobacterium breve* BR03) experienced a significant improvement in clinical parameters [58]. This improvement was associated with enhanced T-helper cell (Th)17/regulatory T cell (Treg) and Th1/Th2; none of these changes were observed in the placebo group [58]. Another study evaluated 109 patients who were randomly divided into four groups: a placebo group, an oligosaccharide group, a *Bifidobacterium bifidum CCFM16* group, and a *Lactobacillus plantarum CCFM8610* group. One of the findings was that ingestion of *Lactobacillus plantarum* CCFM8610 significantly improved atopic dermatitis and increased serum IL-10 levels [59].

We also observed modulation of the intestinal barrier through the expression of the genes *Tjp1*, *Athoh1*, and *Muc2*. The induction of ACD led to a reduction in the expression of *Tjp1* and *Atoh1*. However, treatment with probiotics in the OXA group resulted in an increase in the expression of these genes, suggesting that the probiotic effectively reversed the alterations caused by the skin disease. These genes play an important role in maintaining the intestinal barrier. *Tjp1* is a gene that encodes the protein ZO-1, which functions to anchor junctional proteins that play a critical role in maintaining the structure and permeability of intestinal cells [60]. Increased intestinal permeability is associated with the pathogenesis of intestinal diseases and a reduction in the ZO-1 protein encoded by *Tjp1* [60,61]. The transcription factor *Atoh1* plays a critical role in the differentiation of stem cells into goblet cells [62,63]. Stabilization of ATOH1 enables the maintenance of goblet cell numbers, and reduced expression of this transcription factor decreases the proliferation of these secretory cells [62,63]. Goblet cells contribute to intestinal homeostasis and host defense by secreting MUC2 and other mucins that form a gel-like mucus layer, preventing bacteria from adhering to the epithelial surface, protecting against physical and chemical injuries, and removing pathogens and toxins through intestinal excretion [63,64]. Reduced secretion results in the development of intestinal inflammations such as colitis and bacterial translocation [63,64]. MUC2, which is associated with intestinal homeostasis, is a protein that forms a barrier near the intestinal epithelium, preventing bacteria from reaching and adhering to it [64,65]. There was a trend toward reduced expression in the OXA group compared to the basal group, suggesting that dermatitis may lead to decreased *Muc2* transcription. Probiotic treatment, on the other hand, showed a trend toward increased *Muc2* expression compared to the OXA group. There are experimental studies showing that acetate and *Lachospiraceae* family can increase *Muc2* expression [66,67]. In clinical studies, there is no evaluation of the expression of these genes associated with dermatitis, but rather an assessment of intestinal permeability. One study conducted with 41 children with moderate to severe allergic dermatitis assessed the intake of a combination of two probiotics (*Lactobacillus rhamnosus* 19070-2 and *Lactobacillus reuteri* DM122466) over six weeks [68]. The results showed a significant reduction in dermatitis severity and the frequency of intestinal symptoms. Additionally, a positive association was observed with the lactose/mannitol ratio, a marker of intestinal permeability [68]. Another study conducted in adults investigated the effects of consuming a combination of two probiotics (*Lactobacillus salivarius LS01* and *Bifidobacterium breve BRO3*) for the treatment of allergic dermatitis in adults [58]. The results showed a significant improvement in clinical parameters compared to baseline, as well as inhibition of bacterial translocation [58]. In summary, the induction of ACD diminished the expression of genes essential for maintaining and repairing the intestinal barrier. Remarkably, probiotic treatment restored the expression of these crucial genes, thereby playing a vital role in preserving intestinal homeostasis.

Considering all the findings above, we evaluated intestinal mucus production. Basic mucus production in the distal portion was reduced in the OXA/saline group compared to the basal group, and the probiotic did not affect this state. The findings indicate that the experimental group with dermatitis exhibits a compromised intestinal barrier. Given the essential role of basic mucus in maintaining intestinal pH, it is likely that this group is experiencing a disruption in pH homeostasis. Moreover, the physical barrier appears to be impaired, thereby rendering the intestine more susceptible to damage and inflammation. Here, we can hypothesize that *Murribaculaceae* in OXA/saline might be degrading the mucus [69]. Although this family is not the only one to degrade intestinal mucus, this association is supported by data obtained on *Muc2* expression. On the other hand, acidic mucus production was the same in both the basal and OXA groups, but probiotic treatment increased this type of mucus. Acidic mucus is essential for preventing intestinal damage caused by pathogens and other insults, and its reduction makes the intestine more vulnerable to lesions, inflammation, and infections [70,71]. The increase in acidic mucus production with probiotic treatment is significant, as acidic mucus plays a crucial role in modulating the microbiota composition [70,71]. It promotes the growth of beneficial bacteria while limiting the proliferation of pathogenic bacteria. In this context, the probiotic supports intestinal defense by enhancing this protective barrier.

Finally, we evaluated whether the probiotic used would influence the ACD. Our data indicated an improvement in edema but not in inflammation within the analyzed timeframe, 24 h after the last challenge. It is possible that different results could be obtained at a later point, as studies have shown the effects of this probiotic on skin diseases [8,72,73].

Although the results are innovative and provide important insights into the skin–gut axis, several gaps remain that our study did not address. For instance, it would be valuable to subject the animals with ACD to additional intestinal stress or stimuli to determine whether they respond differently from the control group. Furthermore, observing intestinal changes over a longer period could offer deeper insights. The effects of the probiotic observed in this study were primarily in a preventive context; therefore, exploring whether probiotics could modify intestinal alterations during disease exacerbation would be an interesting avenue for future research. It is also important to note that, in this study, we did not use a cervical collar (Elizabethan collar) to prevent the animals from grooming themselves, which could have led to the ingestion of the agents applied to their skin. While the statistical requirements were met, the number of experimental animals was limited. Together, these limitations highlight important directions for future research.

## 5. Conclusions

The data obtained suggest that skin lesions induced by ACD may cause significant alterations in the gut microbiota, as well as increase the expression of IL-6 and reduce the expression of genes associated with intestinal barrier integrity, also affecting the intestinal mucus. These results indicate a possible predisposition to the development of inflammation in the gut in individuals with ACD, which could be further understood and explored through exposure to specific agents to more precisely evaluate the effects of this intestinal inflammation.

The administration of the probiotic BL showed beneficial effects, improving most of the intestinal parameters evaluated, suggesting that modulating the gut microbiota through probiotics could play a therapeutic role in preventing intestinal diseases associated with skin conditions in humans. These findings highlight the importance of probiotic intervention as a potential preventive strategy for managing intestinal disorders related to skin diseases, such as ACD, which have a significant impact on the overall health of patients.

## Figures and Tables

**Table 1:** IndVal species indicators at ASV level. The multipatt function from indicspecies package was used with a *p* value of 0.05 and 999 permutations.

Naïve			OXA/Saline			OXA/BL		
Taxon	IndVal Stat	*p* Value	Taxon	IndVal Stat	*p* Value	Taxon	IndVal Stat	*p* Value
p__Firmicutes|g__[Eubacterium] brachy group|ASV233	0.914	0.018	p__Bacteroidota|f__Muribaculaceae|ASV83	0.934	0.004	p__Firmicutes|o__Clostridia UCG-014|ASV200	0.989	0.001
p__Firmicutes|f__Erysipelotrichaceae|ASV160	0.879	0.033	p__Firmicutes|g__Lachnospiraceae NK4A136 group|ASV99	0.916	0.010	p__Firmicutes|f__Lachnospiraceae|ASV344	0.956	0.001
p__Firmicutes|f__Ruminococcaceae|ASV214	0.833	0.042	p__Bacteroidota|f__Muribaculaceae|ASV33	0.916	0.012	p__Bacteroidota|f__Muribaculaceae|ASV83	0.934	0.004
p__Firmicutes|f__Lactobacillaceae|ASV283	0.779	0.029	p__Bacteroidota|f__Muribaculaceae|ASV97	0.914	0.001	p__Bacteroidota|f__Muribaculaceae|ASV33	0.916	0.012
			p__Firmicutes|g__[Eubacterium] siraeum group|ASV113	0.903	0.031	p__Firmicutes|g__[Eubacterium] brachy group|ASV233	0.914	0.018
			p__Bacteroidota|f__Muribaculaceae|ASV91	0.901	0.002	p__Firmicutes|o__Clostridia UCG-014|ASV184	0.900	0.006
			p__Firmicutes|o__Clostridia UCG-014|ASV184	0.900	0.006	p__Firmicutes|g__Tuzzerella|ASV435	0.897	0.004
			p__Firmicutes|g__Lachnospiraceae NK4A136 group|ASV54	0.878	0.002	p__Bacteroidota|f__Muribaculaceae|ASV108	0.894	0.002
			p__Firmicutes|g__Lachnospiraceae NK4A136 group|ASV138	0.858	0.045	p__Firmicutes|f__Erysipelotrichaceae|ASV160	0.879	0.033
			p__Patescibacteria|g__Candidatus Saccharimonas|ASV251	0.830	0.019	p__Firmicutes|f__Lachnospiraceae|ASV164	0.861	0.004
			p__Firmicutes|g__Marvinbryantia|ASV298	0.793	0.011	p__Firmicutes|g__Lachnospiraceae NK4A136 group|ASV138	0.858	0.045
			p__Firmicutes|f__Lachnospiraceae|ASV325	0.758	0.031	p__Firmicutes|g__Lachnospiraceae NK4A136 group|ASV116	0.840	0.028
			p__Firmicutes|o__Clostridia UCG-014|ASV156	0.756	0.027	p__Firmicutes|f__Ruminococcaceae|ASV214	0.833	0.042
			p__Firmicutes|g__Lachnospiraceae NK4A136 group|ASV217	0.744	0.012	p__Patescibacteria|g__Candidatus Saccharimonas|ASV251	0.830	0.019
			p__Firmicutes|g__Lachnospiraceae UCG-010|ASV542	0.715	0.014	p__Bacteroidota|f__Muribaculaceae|ASV249	0.829	0.004
			p__Bacteroidota|g__Bacteroides|ASV237	0.695	0.031	p__Firmicutes|g__Oscillibacter|ASV336	0.819	0.005
			p__Firmicutes|f__Ruminococcaceae|ASV335	0.691	0.036	p__Firmicutes|f__Oscillospiraceae|ASV294	0.815	0.013
						p__Bacteroidota|f__Muribaculaceae|ASV252	0.792	0.009
						p__Firmicutes|f__Ruminococcaceae|ASV293	0.788	0.016
						p__Firmicutes|f__Lachnospiraceae|ASV235	0.781	0.017
						p__Bacteroidota|f__Muribaculaceae|ASV189	0.779	0.019
						p__Firmicutes|f__Lachnospiraceae|ASV255	0.775	0.008
						p__Firmicutes|f__Lachnospiraceae|ASV449	0.775	0.013
						p__Firmicutes|g__Lachnoclostridium|ASV459	0.775	0.013
						p__Actinobacteriota|g__Enterorhabdus|ASV340	0.770	0.017
						p__Firmicutes|g__Lachnospiraceae NK4A136 group|ASV241	0.761	0.026
						p__Firmicutes|o__Clostridia UCG-014|ASV156	0.756	0.027
						p__Bacteroidota|f__Muribaculaceae|ASV285	0.731	0.015
						p__Firmicutes|g__Lachnospiraceae FCS020 group|ASV354	0.729	0.022
						p__Firmicutes|g__Lachnospiraceae NK4A136 group|ASV179	0.727	0.011
						p__Firmicutes|f__Lachnospiraceae|ASV359	0.710	0.019
						p__Firmicutes|g__Lachnoclostridium|ASV300	0.709	0.016
						p__Firmicutes|g__Lachnospiraceae NK4A136 group|ASV229	0.707	0.02
						p__Firmicutes|g__GCA-900066575|ASV564	0.706	0.032
						p__Firmicutes|f__Lachnospiraceae|ASV410	0.699	0.034
						p__Firmicutes|f__Christensenellaceae|ASV496	0.697	0.031

## Data Availability

The original contributions presented in this study are included in the article/Supplementary Material. Further inquiries can be directed to the corresponding author.

## Supplementary Materials

The following supporting information can be downloaded at: https://www.mdpi.com/article/10.3390/microorganisms13051082/s1, Figure S1. Protective effects of prophylactic oral supplementation with *Bifidobacterium longum* strain BB536 on ear inflammation in a murine model of ACD. Animals were pretreated with probiotic, sensitized, and subsequently challenged with oxazolone (OXA). Twenty-four hours post-challenge, the following parameters were analyzed: (A) Photomicrograph of H&E-stained ear tissue sections captured using a 100× objective. (B) Ear thickness measured with a digital external micrometer. (C) Total leukocyte infiltration in ear tissues of ACD mice. Statistical analysis was performed using Student’s *t*-test. Data are presented as mean ± SEM. Statistical significance is considered as *p* < 0.05. Figure S2. Effects of Contact Dermatitis and Probiotic Treatment on Cytokine Levels in the Colon. Mice were pretreated with the probiotic (OXA/BL; n = 7) or saline (OXA/Saline; n = 7), sensitized, and challenged with oxazolone (OXA). The naïve group (n = 7) was not subjected to any treatment. Twenty-four hours post-challenge, the colon was collected and analyzed by ELISA to quantify (A) IL-6, (B) IL-13, and (C) IL-33 levels. ND: Not detectable (below the detection limit). Statistical analysis was performed using one-way ANOVA. Data are presented as mean ± SEM. Statistical significance is considered as *p* < 0.05. Figure S3: Indicator taxa with significant *p*-values in the IndVal test for each treatment group. The *x*-axis represents the IndVal statistic, which quantifies the association strength between taxa and treatment groups. Bars are color-coded according to the group in which the taxa were identified as indicators: Naive (dark blue), OXA/Saline (cyan), and OXA/BL (red).
