# Supplementary material for: Probiotic Administration Contributes to the Improvement in Intestinal Dysregulation Induced by Allergic Contact Dermatitis
Source: Microorganisms. 2025 May 7;13(5):1082. doi: 10.3390/microorganisms13051082 (PMC12114202; doi:10.3390/microorganisms13051082)
Supplement: Supplementary file 1 [file microorganisms-13-01082-s001.zip › microorganisms-3559233-supplementary.pdf]

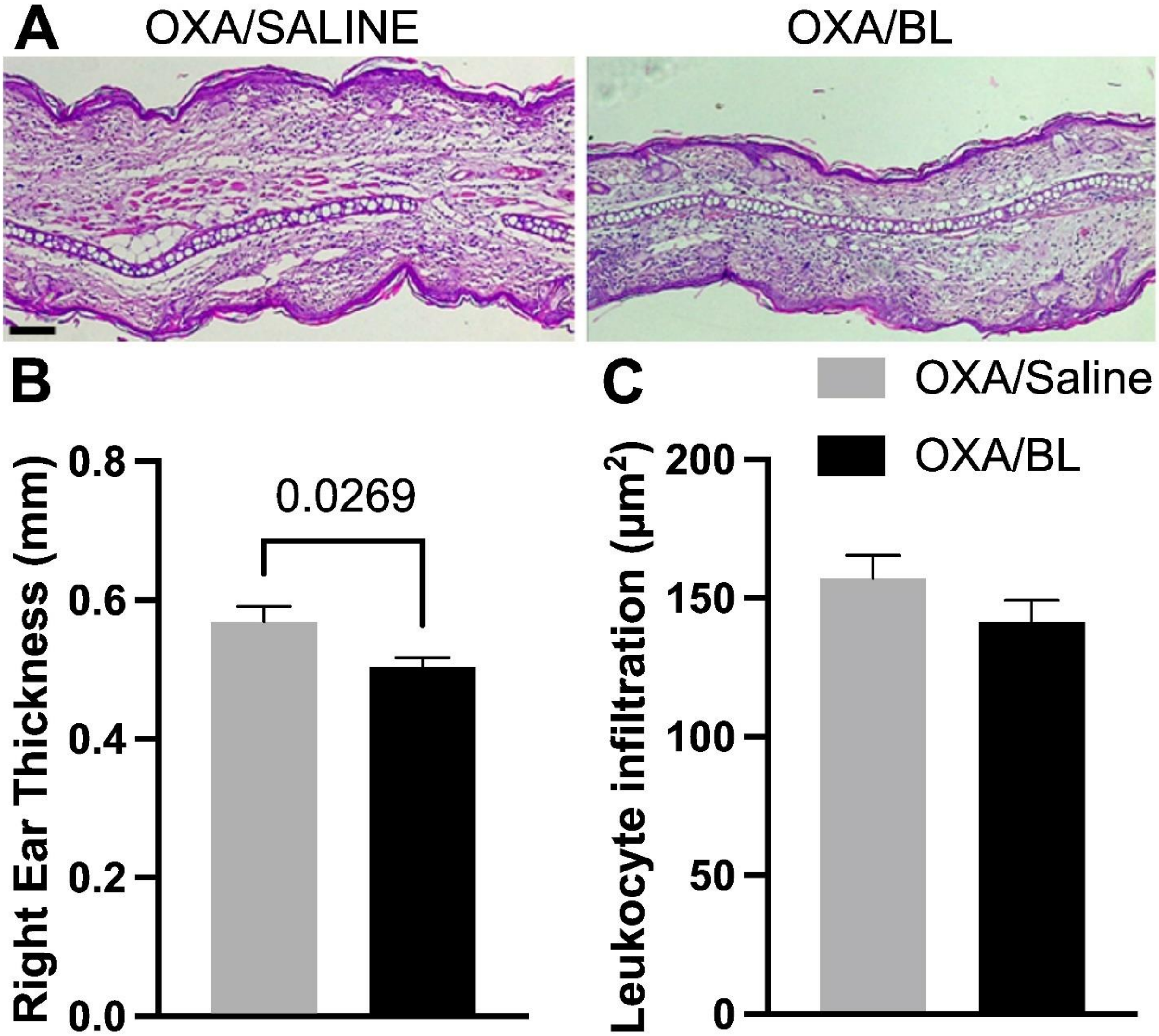

Figure S1. Protective effects of prophylactic oral supplementation with *Bifidobacterium longum* strain *BB536* on ear inflammation in a murine model of ACD. Animals were pretreated with probiotic, sensitized, and subsequently challenged with oxazolone (OXA). Twenty-four hours post-challenge, the following parameters were analyzed: (A) Photomicrograph of H&E-stained ear tissue sections captured using a 100x objective. (B) Ear thickness measured with a digital external micrometer. (C) Total leukocyte infiltration in ear tissues of ACD mice. Statistical analysis was performed using Student's T-test. Data are presented as mean ± SEM. Statistical significance is considered as $p < 0.05$.

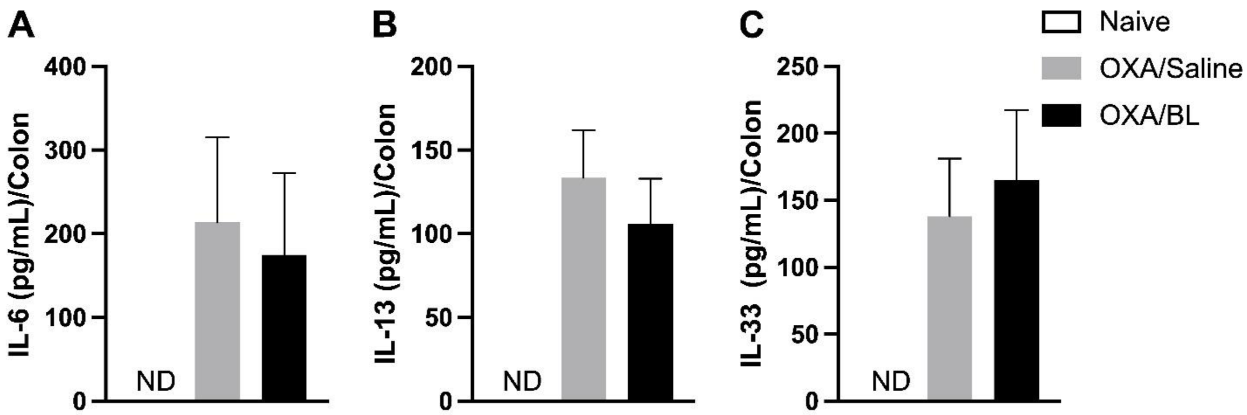

Figure S2. Effects of Contact Dermatitis and Probiotic Treatment on Cytokine Levels in the Colon. Mice were pretreated with the probiotic (OXA/BL; n=7) or saline (OXA/Saline; n=7), sensitized, and challenged with oxazolone (OXA). The naïve group (n=7) was not subjected to any treatment. Twenty-four hours post-challenge, the colon was collected and analyzed by ELISA to quantify (A) IL-6, (B) IL-13, and (C) IL-33 levels. ND: Not detectable (below the detection limit). Statistical analysis was performed using one-way ANOVA. Data are presented as mean ± SEM. Statistical significance is considered as $p < 0.05$.

)

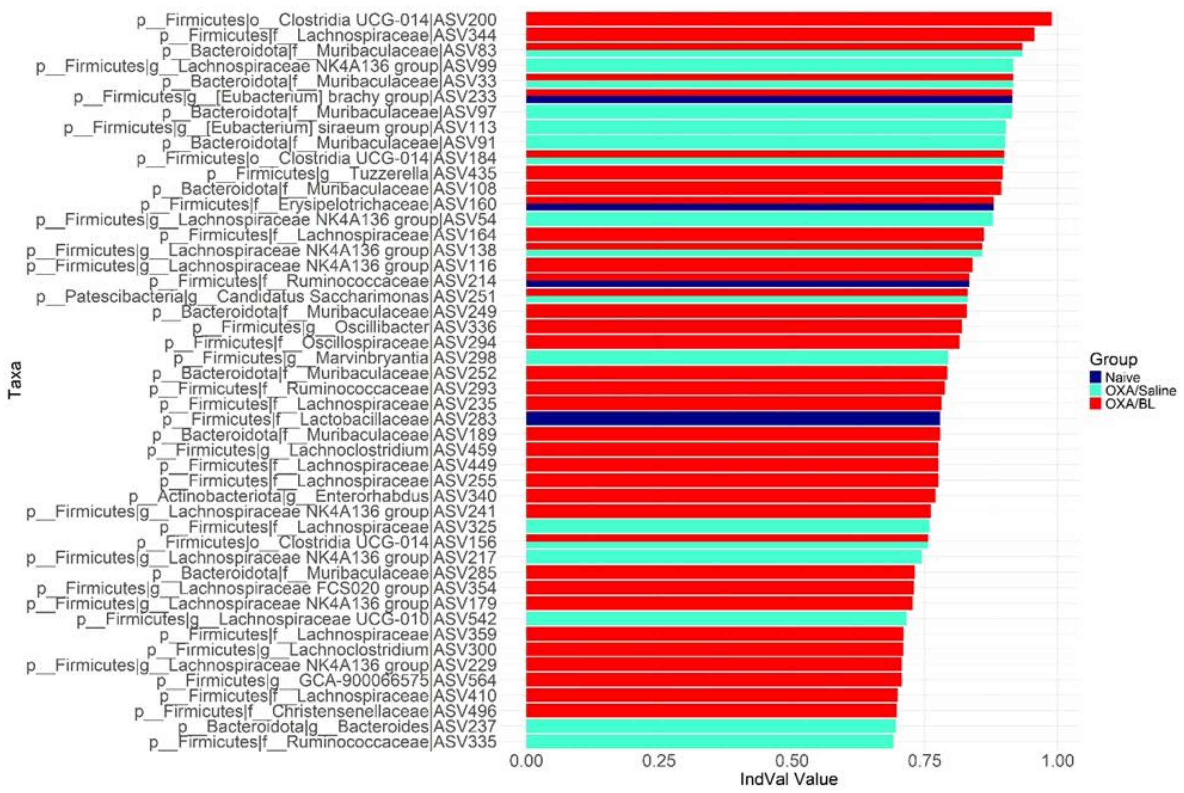

Supplementary Figure S3: Indicator taxa with significant p-values in the IndVal test for each treatment group. The x-axis represents the IndVal statistic, which quantifies the association strength between taxa and treatment groups. Bars are color-coded according to the group in which the taxa were identified as indicators: Naive (dark blue), OXA/Saline (cyan), and OXA/BL (red).
